# An Ingestible Electronics for Continuous and Real-Time Intraabdominal Pressure Monitoring

**DOI:** 10.3390/jpm11010012

**Published:** 2020-12-24

**Authors:** Chien-Hung Liao, Chi-Tung Cheng, Chih-Chi Chen, Uei-Ming Jow, Chun-Hung Chen, Yen-Liang Lai, Ya-Chuan Chen, Dong-Ru Ho

**Affiliations:** 1 Department of Trauma and Emergency Surgery, Linkou Chang Gung Memorial Hospital, Chang Gung University, Taoyuan 333423, Taiwan; surgymet@gmail.com (C.-H.L.); atong89130@gmail.com (C.-T.C.); jow0209@gmail.com (U.-M.J.); snoobe@gmail.com (Y.-L.L.); joycechen108@gmail.com (Y.-C.C.); 2 Department of Rehabilitation and Physical Medicine, Linkou Chang Gung Memorial Hospital, Chang Gung University, Taoyuan 333423, Taiwan; claudia5477@gmail.com; 3 Department of Chemical Engineering, National United University, Miaoli 360, Taiwan; brucechen@advbiomedtech.com; 4 Department of Urology, Chiayi Chang Gung Memorial Hospital, Chang Gung University, Chiayi 613016, Taiwan

**Keywords:** intraabdominal hypertension, abdominal compartment syndrome, smart capsule, wearable device

## Abstract

Abdominal compartment syndrome can be treated through decompressive surgery if intraabdominal hypertension (IAH) can be detected in time. Treatment delays due to manual, conventional intravesical pressure (IVP) monitoring using a Foley catheter have been reported. In this work, we present an innovative gastrointestinal intraluminal pressure (GIP) measurement-based method to monitor and improve pressure-guided relief of intraabdominal pressure (IAP). A novel algorithm for detecting IAH in the gastrointestinal tract of a live porcine model is reported. A wireless pressure-sensing capsule (10 × 13 mm) was developed for absolute measurement. The IAP was estimated during artificial pneumoperitoneum. The pressure waveform-based measurements indicated that the wireless pressure sensor could be used to predict IAP. To enhance GIP monitoring for predicting IAH, the proposed continuous ingestible wireless electronics-based pressure waveform measurement device can be used as a complement to existing modalities. The use of the proposed pressure measurement and communication technology can help provide valuable data for digital health platforms.

## 1. Introduction

Intraabdominal hypertension (IAH) can cause organ dysfunction in patients who have been treated in intensive care, surgery, trauma, and cardiology departments. It is also increasingly recognized in patients after elective surgical procedures, including liver transplantation, massive fluid resuscitation for extra-abdominal trauma, severe burns, and aortic aneurysm repair [1,2,3]. Since the early 2000s, abdominal compartment syndrome (ACS) has been accepted as a well-defined clinical entity. Monitoring intraabdominal pressure (IAP) is mandatory, particularly in critically ill patients in intensive care units [4]. Moreover, given the risk factors that could lead to an increase in IAP and progression to ACS, IAP monitoring has made it possible to detect early signs of IAH in patients under treatment [5]. The early detection of ACS is key to obtaining the best treatment results [6]. In 2006, the World Society of Abdominal Compartment Syndrome (WSACS) defined IAH as IAP elevation above 12 mm Hg in three consecutive measurements [7]. Furthermore, ACS is defined as an IAP above 20 mm Hg and in conjunction with associated organ dysfunction or failure [8,9].

Early recognition of ACS from its clinical signs and risk factors can significantly reduce the associated morbidity and mortality. If clinical signs can be detected in time, emergent decompressive laparotomy can be performed immediately to relieve the pressure [10,11]. Because early signs are easily ignored, the patient mortality rate can reach as high as 55% if ACS occurs [6,12,13]. Therefore, IAP monitoring is an important clinical step in the intensive care of critical abdominal diseases [14].

The gastrointestinal (GI) tract offers an opportunity to detect pathophysiological signals from the human body. Ingestible electronics that are capable of embedded sensing can achieve close proximity to major organs through the GI tract, can serve as clinical tools for diagnostics and have the potential to screen for diseases that are difficult if not impossible to detect at an early stage using other means [15,16]. These technological advances have the potential to make disease surveillance and treatment far more effective for a variety of conditions, allowing patients to lead longer and more productive lives [16]. Ingestible electronic devices are used in a number of applications [15,17,18,19], including capturing video [20], tracking patient compliance [21], sensing chemical composition [22,23], reading internal temperature, measuring pH, and timing motility [24,25]. However, no study has yet investigated the application of ingestible electronic systems for the detection of IAP. In this study, we developed a wireless intragastrointestinal pressure sensing system that can detect the IAP and evaluated the possibility of diagnosing IAH through wireless continuous monitoring in animal models.

## 2. Materials and Methods

### 2.1. Device Design

**Figure 1:**
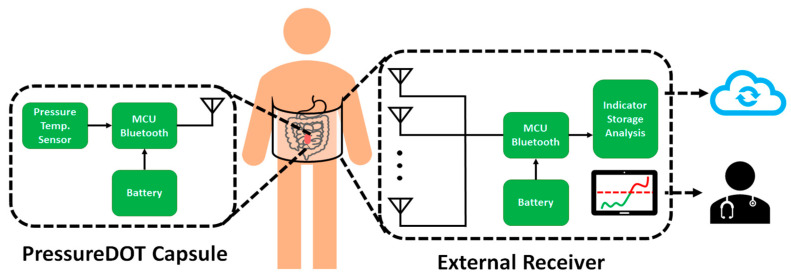
Illustration of the wireless sensor system, PressureDot, which is composed of two parts, an ingestible electronic capsule and a external receiver, that detect continuous changes in intraabdominal pressure in real time. After the external receiver obtains the signal from the capsule, the data can be sent to health providers and is collected in a cloud-based databank.

Our proposed PressureDot (PDT) pressure sensing system includes a single-use capsule sensor, which consists of a wireless pressure sensor with a diameter (D) of 10 mm and a length (L) of 13 mm. The capsule can be swallowed, remains in the gastrointestinal tract for continuous IAP measurement and is excreted within 72 to 120 h. The PDT system also includes an ultralow power-consuming special application integrated circuit with memory, a microelectromechanical system (MEMS) pressure sensor, and a miniature foldable Bluetooth antenna to send signals to external devices. The detectable pressure range is –100 mm Hg to 100 mm Hg under 1 atmosphere (ATM) within an accuracy of ±0.75 mm Hg. The PDT transmits pressure signals in real time through the Bluetooth Low Energy (BLE) 5.1 protocol. The encrypted broadcasting data is transmitted every 10 s (0.1 Hz); the communication is one way, meaning that the device will not receive any signals from any external Bluetooth devices. The capsule has sufficient battery power to ensure more than 144 h of operation. The system function block is shown in Figure 1.

The internal device is a wireless pressure sensor, and the external receiver includes an antenna array, microcontroller (MCU), memory, and indicator. The indicator can display the real-time pressure measurements to health care providers. The data can be uploaded to the cloud long term to provide valuable digital health information.

### 2.2. Fabrication

**Figure 2:**
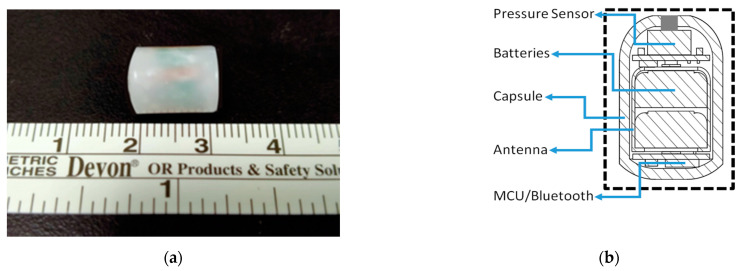
(**a**) The PressureDot ingestible wireless pressure sensing capsule, measuring 13 mm in length and 10 mm in diameter. (**b**) Illustration of the ingestible wireless pressure sensing capsule, composed of a biocompatible pressure sensor with a flexible antenna for wireless monitoring and recording of gastrointestinal intraluminal pressure and intraabdominal pressure.

The support structure and housing of the capsule were fabricated from a biocompatible polyester-based resin (polycaprolactone, MeDFila CL01A, Advanced Biomedical Technology) in a class 7 environment to maintain a high quality. The inner support framework was assembled with a rigid-flex printed circuit board assembly (PCBA) composed of batteries and a pressure transducer in a guide-groove insert to form the system module. Before the module was encapsulated, deposition was performed, followed by atomic layer deposition (ALD) of platinum (40 nm) to protect the pressure sensor. The system module was assembled manually and then cast into a capsule structure (polylactic acid, PLA). To ensure that no air pockets were trapped in the assembled capsule, the encapsulated device was postcured in its housing in a low-temperature oven (45 °C) at low pressure (600 mm Hg) for 10–20 min. Holes were opened at the top of the capsule (diameter = 500 μm) to allow pressure transmission to the transducer. The complete PDT capsule is shown in Figure 2.

### 2.3. In Vivo Study

**Figure 3:**
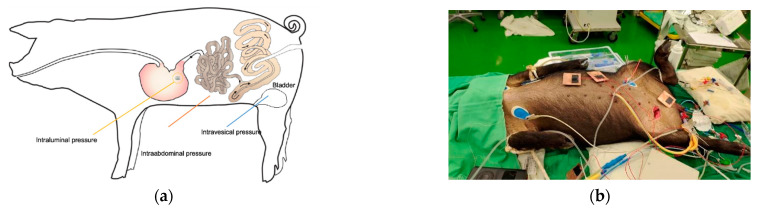
(**a**) Illustration of the pressure detection method used in this swine study. Intraabdominal pressure was created and manipulated by pneumoperitoneum. Then, we inserted one pressure probe into the porcine bladder to measure the intravesical pressure, one probe into the peritoneal cavity to measure the intrabdominal pressure, and a third ingestible pressure sensor into the porcine gastrointestinal tract to measure the intraluminal pressure. (**b**) Photograph of the animal study. The pressure detectors settled well into the porcine cavities, and wireless data was collected with external antenna receivers.

An in vivo animal study was conducted to demonstrate the consistency between intravesical (IVP), intraabdominal, and gastrointestinal intraluminal pressure (GIP) data obtained with invasive and noninvasive pressure sensing devices. The experimental setup for inserting the wireless sensor into the gastrointestinal intraluminal space of a porcine model using a high-resolution wireless pressure sensing analyzer is shown in Figure 3.

The experimental animals were under the care of the Chang Gung Memorial Animal Care Center. All animal studies were carried out using the protocols of the Laboratory Animal Care and Use Committee of the Chang Gung Memorial Hospital (Animal experiment approval number: 29062501) and adhered to the National Institutes of Health (NIH) guidelines for ethical animal research. Female domestic Lanyu pigs of gastrointestinal maturity (approximately 9–15 months of age) were used in this study.

For the live porcine animal model, a 9-month-old female pig with a weight of ~30 kg was procured. The room temperature for the animal experiments was adjusted to 22 °C to control the influence of temperature on the pressure value. The porcine received general anesthesia using 1:1 *v*/*v*% zolazepam, tiletamine and xylazine hydrochloride injected into the muscle at 0.1 mL/kg. During the whole experiment, an anesthetic plane was maintained using a respiratory anesthesia device (isoflurane) to keep the animal sedated for sufficient durations and to prevent movements. We did not paralyze the animals in order to prevent changing the muscle tone of the abdominal wall and GI tract in the porcine model. The hair of the abdomen was removed using surgical blades, and the outer skin surfaces were wiped with 7% ethanol.

The pressure was measured using a piezoelectric-based pressure monitor, and the measured value was reported through a customized antenna-receiver graphical user interface (GUI). Our wireless pressure device (PressureDot Technology, Inc., Chiayi County, Taiwan) was used as the ingestible wireless electronics. The ingestible wireless electronics device was sealed to be waterproof sealed except for the sensor channel. Before ingestion, the abdomen was scanned using radiography, and the time-zero bowel condition was determined. Smooth forceps and a laryngoscope were used to insert the wireless device beyond the epiglottis to prevent airway obstruction and achieve successful ingestion. The wireless electronics later passed through the esophagus into the stomach, initiating the journey in the gastrointestinal intraluminal space. The change in the intraluminal pressure was monitored continuously in real time. The highest point in the frequency range of the current system was 100 Hz, and the sampling rate was 33 signal/s. The pressure spectrum was measured at a frequency of 100 Hz. After the wireless electronics device was inserted into the gastrointestinal intraluminal space, the pressure was measured as it passed down via peristalsis.

**Figure 4:**
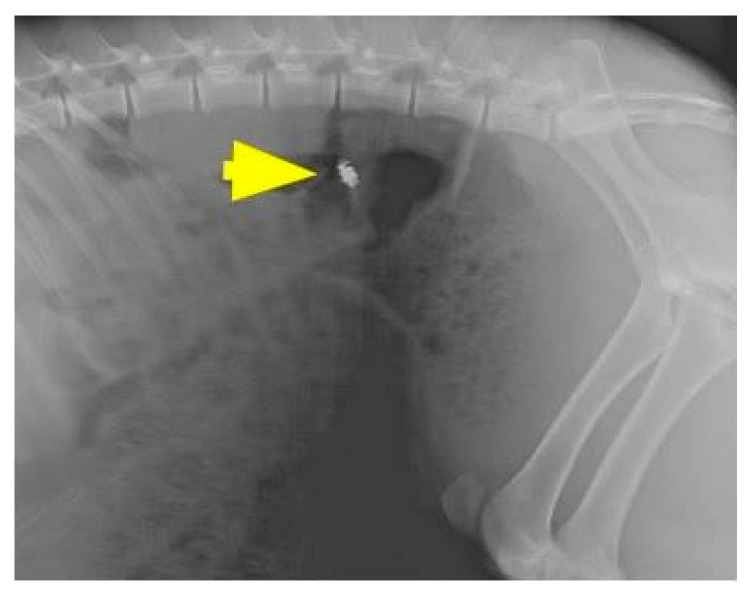
The location of the ingestible capsule, PressureDot, was checked using fluoroscopy. Arrow: the ingestible capsule.

Pressure sensors (telemetric pressure probes and/or PDT) were inserted into each animal, as illustrated in Figure 3. Following the insertion of gastrointestinal intraluminal pressure (GIP)-sensing devices, herein called PDT, the animals were observed for 72 h to monitor for wireless sensor capsule operation, leakage, embolic events, or other adverse events as they related to the surgical procedure. The location of the wireless electronics was checked using fluoroscopy (Siemens Inc., Munich, Germany), as shown in Figure 4.

**Figure 5:**
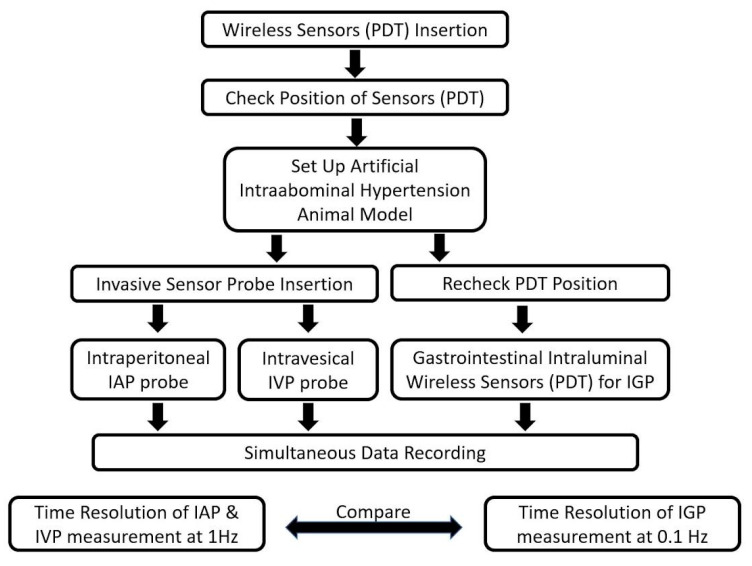
Flowchart of the in vivo study.

If the PDT reached the stomach of porcine, we anesthetized the swine and inserted a telemetric pressure probe. All relevant parameters (ventilation and blood pressure) and IAP measurements were first documented at baseline IAP in the supine position. An artificial IAH porcine model was created by inducing pneumoperitoneum with a carbon dioxide insufflator (Endoflator, Karl Storz, Tuttligen, Germany) [26,27], which is used in conventional laparoscopic operations, and IAH was simulated when the IAP reached over 20 mm Hg. A Veress needle (Karl Storz, Tuttlingen, Germany) was placed intraperitoneally through a 0.5 cm skin incision directly caudal to the umbilicus. CO_2_ was insufflated until an IAP plateau at the target pressure level was reached in the first step by the insufflator. After induction of pneumoperitoneum, measurements were obtained again 2 min after reaching the IAP target plateau at 8 defined pressure points for 10 min given by the Endoflator and shifted to the next level in a two-minute interval. This standard article will be referred to as IAP. The mean value of these eight repeated measurements was used for analysis. Animals were euthanized after decompression of the capnoperitoneum. The details of this study are illustrated in Figure 5.

## 3. Results

**Figure 6:**
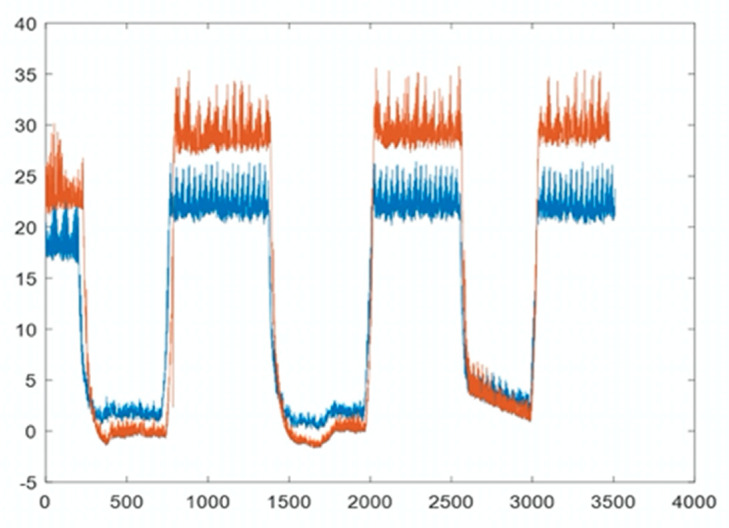
Relationship between the intravesical pressure detected by the pressure probe (brown) and PressureDot (blue) showing a with high correlation, R = 0.9498.

In this study, we demonstrated that the PDT can consistently measure pressure values comparable to invasive measurements (telemetric pressure probe) that served as a control to provide the absolute pressure in the bladder. Figure 6 below shows the IVP measured between the PDT (brown) and the invasive pressure probe (blue). These pressure measurements were linearly correlated (R = 0.9498), and a trend of pressure increase was also observed during the simulated IAH.

Furthermore, the pressure data collected from the three swines with the PDT, intraperitoneal pressure probe, and intravesical pressure probe were compared. As outlined in Table 1, the GIP was measured by the PDT, while the IAP and IVP served as the controls to provide ground-truth measurements using telemetry pressure probes (via invasive insertion).

**Figure 7:**
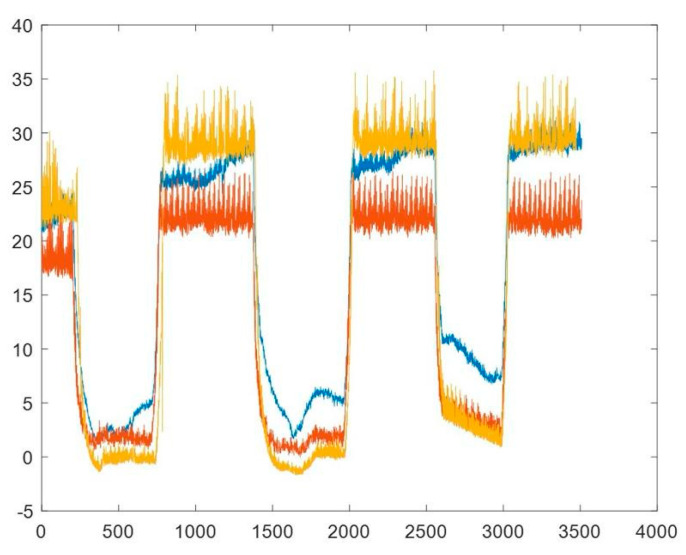
The pressure wave changes in the normal pressure phase to intraabdominal hypertension phase recorded intraluminally from the PressureDot (yellow line), intravesically from the pressure probe (brown line) and intraabdominally from the intraperitoneal pressure probe (blue line).

The pressure measurement waveforms are shown in Figure 7. The baseline intrabdominal pressure was 0 mm Hg and we began to increase pressure with carbon dioxide inflation. The IVP (brown) measured between the PressureDot (yellow) and the invasive pressure probe (blue) were linearly correlated, and a trend of increasing pressure was also observed during normal IAP to the simulated IAH.

The data demonstrated that the PressureDot can consistently measure pressure values comparable to invasive measurements (telemetric pressure probes) that served as controls to provide the absolute ground-truth pressure in the bladder and in the peritoneal cavity, as shown in Table 1. The IAP and GIP were comparable to each other. However, the IVP was generally lower than the other two pressure measurements.

## 4. Discussion

In this study, our team developed an ingestible electronic device for continuous IAP monitoring called PDT that can directly measure IAP in real time. It can also provide pressure trends and, potentially, other clinical information to determine perfusion pressure within the intraabdominal organs to facilitate making correct medical decisions. In a comparison with current direct and indirect measurement methods for IAP, including telemetric piezoresistive probes at pelvic sites for direct IAP measurement and intragastric and intravesical probes for indirect IAP measurement, the PDT showed comparable diagnostic accuracy. Simulated IAH ranging from 10 to 40 mm Hg was introduced, including the range of ACS (20 to 40 mm Hg), and the relative pressures were measured and compared. The results showed that all the devices, including the PDT, could detect elevated abdominal pressures. The performance of the PDT was comparable to that of currently available instruments with a good correlation (R = 0.9498)

The abdominal compartment normally sustains a pressure of approximately 5–7 mm Hg [28], and many pathologic conditions can generate sustained pressures greater than 12 mm Hg [7], a state referred to as IAH, producing subclinical organ dysfunctions leading to multiple organ dysfunction syndromes [5]. Thus, ACS is seen as the result of a sustained IAH [29] Increased attention to IAP, along with changes in the clinical management of critically ill or injured patients, has led to exponential growth in research relating to IAH and ACS in recent years. Milestones have included the incorporation of the WSACS and the Societies’ publication of IAH and ACS expert consensus definitions in 2006 [30], and the epidemiology and management became consistent. Trigger factors located inside the abdominal cavity can induce primary ACS, whereas those outside of the abdominopelvic cavity can contribute to the development of secondary ACS. Currently, IVP monitoring is considered the acceptable measurement route [5,7,31]. Although the benefits of IVP monitoring in the diagnosis, prevention, and management of IAH have been demonstrated, some clinicians remain reluctant to institute this monitoring technique out of concern for increasing the patient’s risk of device-related nosocomial urinary tract infection while potentially altering the aseptic condition of the urinary drainage system [32]. In addition to possible nosocomial infections, the laborious loading and high time consumption are considered other limitations. The traditional measurement is often conducted once every 4–6 h, typically not the optimal period for monitoring the IAH/ACS progress [5,7,33]. Continuous instillation of saline into the urinary bladder can increase the possibility of vesicoureteral reflux, which is another limitation of IVP monitoring. Therefore, the development of additional interfaces is critical for monitoring the IAP of critical patients.

The PDT takes a targeted approach in the development of a wireless real-time IAP measurement system, capable of measuring pressures ranging from 0 to 40 mm Hg. The potential benefits include a reduced ICU stay for early IAH detection, decreased ACS-related mortality, and a reduced medical financial burden [34] Assessment of IAH is rarely performed through intraluminal pressure monitoring because of inadequate methodology and lack of knowledge about the relationship between pressure patterns [35]. In this study, we assessed the correlation between intraluminal pressure and IAP and obtained acceptable results. Unlike other invasive measurements, ingestible electronic devices can potentially reduce labor and promote research for discovering new physiologic parameters for associated pathologies and lead to the development of autonomous diagnostic aids for users [15]. The PDT addresses the major shortcomings of the current intravesical monitoring system, including the inability to archive continuous data, wasted manpower, and the induced vulnerability to urinary tract infection. Several animal models were developed to create intrabdominal hypertension [36,37,38,39,40]. In order to develop the adjustable intraabdominal pressure environment, we used a porcine model with inducing pneumoperitoneum with a carbon dioxide insufflator. Compared with other models, this one can help us to adjust the pressure correctly and, in a time-saving method.

Although this study indicated that the wireless digital capsule can detect the GIP which is comparable with IAP, there are still some limitations. The number of pigs included in the study was limited, which might reduce the power of its results. Although the size of the PDT size is half that of other capsular devices, personal adaption was another consideration. Most the patients who suffer from IAH need ventilator support, the application of the device in the intubated patient is another challenge. Specially designed tool should be used to deliver PDT. The clogged sensor might be another consideration in gastrointestinal intraluminal devices, although we did not have the issue in this study. The respiratory influence on the intraabdominal pressure is another critical parameter. However, we did not have the data to synchronize these waveforms together by the limitation of the instrument. Finally, this study is a pilot study for the validation of the PDT. However, further clinical trials in humans must be performed under the evaluation of an institutional review board (IRB) and after regulatory registration of the products.

## 5. Conclusions

The pilot animal study demonstrated that abdominal pressure can be consistently represented by using either invasive or noninvasive pressure sensing devices. The pressure data from PDT, IGP, and IVP were relatively comparable and had a linear correlation in representing the IAP trend, particularly during the simulated IAH. Continuous monitoring of a patient’s physical status is one goal of health care, especially for critical care, which can help clinical physicians make an immediate response to help the critical disease on time. Moreover, the data will be analyzed based on a new algorithm for the digital health platform that clinical physicians could make an early decision. In short, the use of PressureDOT can improve efficiencies, increase access, reduce costs, decrease risk, and increase quality for abdominal compartment syndrome.

## Figures and Tables

**Table 1:** The average pressure data from different measuring tools under pneumoperitoneum pressure.

Pneumoperitoneum Setting Pressure	IAP	GIP (PDT)	IVP
10 mm Hg	11.52 + 0.06	10.18 + 3.51	11.84 + 0.35
20 mm Hg	20.00 + 0.06	19.86 + 2.20	18.28 + 2.08
30 mm Hg	30.21 + 0.33	31.33 + 2.55	26.21 + 8.71
40 mm Hg	40.06 + 0.09	40.59 + 0.61	43.12 + 1.21

IAP: Intraabdominal pressure, IVP: Intravesical pressure, IGP: Gastrointestinal intraluminal pressure.

## Data Availability

The data presented in this study are available on request from the corresponding author. The data are not publicly available due to the restriction of local law and government policy.
